# Regional Homogeneity Changes in Nicotine Addicts by Resting-State fMRI

**DOI:** 10.1371/journal.pone.0170143

**Published:** 2017-01-12

**Authors:** Hongbo Chen, Shaofeng Mo

**Affiliations:** 1 School of Life and Environmental Sciences, Guilin University of Electronic Technology, Guilin, Guangxi, China; 2 School of Geosci and Info-physics, Central South University, Changsha, Hunan, China; Institute of Psychology, Chinese Academy of Sciences, CHINA

## Abstract

**Objective:**

To reveal the brain functional changes of nicotine addicts compared with those of non-smokers and explore the objective biomarker for nicotine dependence evaluation.

**Methods:**

A total of 14 smokers and 11 non-smoking controls were recruited for this study. Resting-state functional magnetic resonance imaging and regional homogeneity (ReHo) were applied in the neural activity analysis. Two-sample t-test was performed to examine the voxel-wise difference between the smokers and the controls. Correlation analysis between the ReHo values and the Fagerstrom Test for Nicotine Dependence (FTND) scores were performed to explore the biomarkers for the clinical characteristics of smokers.

**Results:**

The ReHo values from the right superior frontal gyrus of the Brodmann’s area (BA) 9 to the right middle frontal gyrus and the ReHo value from the left and right precuneus (BA 23) to the left and right middle cingulum gyrus were lower in the smokers than in the non-smokers. The ReHo value in the precuneus (BA 23) was significantly and positively correlated with the FTND score of smokers.

**Conclusion:**

The ReHo values in the right superior frontal gyrus and left precuneus can be used to separate the smokers from the non-smokers. In particular, the left precuneus is a potential neuroimaging biomarker for nicotine addicts.

## Introduction

Smoking causes many kinds of diseases, such as ischemic heart disease, cerebral vascular disease, lower respiratory tract infection, chronic obstructive pulmonary disease, tuberculosis, and lung cancer [1]. Nicotine addicts often experience psychological and emotional changes [2, 3]. Do et al. [4] discovered that functional coupling between striatal and cortical regions is associated with increased craving in adolescent smokers. Sutherland et al. [5] observed that smoking is associated with convergent structural decreases in the left insula, right cerebellum, parahippocampus, multiple prefrontal cortex regions, and the thalamus. Mcclernon et al. [6] provided evidence that abstinence-induced deficits in working memory and changes in the underlying brain function are mostly due to abstinence from nicotine rather than non-nicotine factors. These findings highlight that smoking addiction is associated with brain function changes.

In the past several years, resting-state functional magnetic resonance imaging (rs-fMRI) has been increasingly used for studying neural activity in the brain. This non-invasive imaging technique offers unbiased analysis of the region-to-region interactions and voxel-by-voxel neural activity at the functional level based on the rudimentary and intrinsic activity of the resting brain [7, 8]. The technology has been widely applied for investigating the brain function and its changes caused by diseases [9–15].

Regional homogeneity (ReHo) is an important research method for rs-fMRI. The method reflects the local temporal homogeneity of the regional blood oxygen level-dependent signal and serves as a complement for brain neural activity in resting state [16]. The change in the ReHo values in the smoking cessation process has been extensively investigated [17–19]. Yu et al. used ReHo combined with fMRI to investigate the spontaneous neural activity in the resting state of heavy smokers [20]; however, they did not analyze the relation between the ReHo values and the clinical characteristics of the participants. The changes in the local synchronization of spontaneous fMRI signals that occur in nicotine addicts during the resting state are rarely explored, as well as the relation between these changes and the clinical characteristics of smokers. Fagerstrom Test for Nicotine Dependence (FTND) is an important index for the clinical evaluation of a smoker’s dependence on nicotine addiction; this index has been widely used in clinical treatment and research on nicotine addiction [21, 22].

In the present study, we hypothesized that the ReHo values of resting-state brain activity would be different between the smokers and the controls, and that the mechanism was related to the FTND scores. The present study aimed to explore the objective biomarker for nicotine dependence evaluation using rs-fMRI.

## Materials and Methods

### Participants

The study was approved by the Ethics Committee of Affiliated Hospital of Guilin Medical University (Guilin, Guangxi, China). A total of 14 smokers and 11 non-smoking controls were recruited for this study. All the participants signed consents to the experiment. The demographic and clinical data of subjects are summarized in Table 1. All participants were healthy males, received 12 years of education or longer, right-handed, showed no contraindications for MRI check, and exhibited no brain structural abnormality.

**Table 1:** Demographic and clinical characteristics of participants in this study.

	Smokers	Controls	p-Value
Characteristics	n = 14,M	n = 11,M	--
Age(year)	34.0±11.7	34.5± 11.0	0.907^a^
Education(years)	16.6±2.7	17.4±3.0	0.537^a^
FTND	5.1±1.5	--	--

^a^ The p-value for the difference between the two groups was obtained by two-sample t-test.

FTND: Fagerstrom Test for Nicotine Dependence. M: Male

All smokers in the study were diagnosed based on the Diagnostic and Statistical Manual of Mental Disorders-IV, and they were smoking more than 10 cigarettes per day for 5 years or longer. The smoking participants had no marijuana, heroin, alcohol, and other drug addiction. They had no previous epilepsy, family history of epilepsy, and other psychiatric disorders. All smokers were tested by FTND.

### Data Acquisition

MRI data were collected with a 3T Siemens Verio MRI scanner at the Affiliated Hospital of Guilin Medical University using a 12-channel head coil. Participants laid in the scanner in the supine position. Cushions and forehead straps were used to immobilize the head to minimize movement. During rs-fMRI data acquisition, participants were instructed to rest with their eyes close.

Functional images were acquired using a single-shot gradient echo-echo planar imaging sequence. The imaging parameters were as follows: field of view (FOV) = 22 cm × 22 cm, slices = 43, slice thickness = 3.2 mm, no slice gap, repetition time (TR) = 2000 ms, echo time (TE) = 30 ms, flip angle (FA) = 90 with nominal resolution = 3.4 mm × 3.4 mm × 3.2 mm and matrix = 64 × 64. The scan time of each rs-fMRI was 480 s, and a total of 240 volumes were collected.

Structural images were acquired using a three-dimensional magnetization- prepared rapid gradient-echo sequence with TR = 1900 ms, TE = 2.52 ms, FA = 8, matrix = 256 × 256, FOV = 25.6 cm × 25.6 cm, slice thickness = 1.0 mm, and no slice gap. A total of 176 slices were used to cover the whole brain.

### Data Analysis

All rs-fMRI data were preprocessed using the software for Data Processing Assistant for Resting-State fMRI (DPARSF, http://www.restfmri.net/forum/DPARSF) based on Statistical Parametric Mapping (SPM8, http://www.fil.ion.ucl.ac.uk/spm) and Resting-state fMRI Data Analysis Toolkit (REST, http://www.restfmri.net) [23, 24].

We converted the DICOM data to NIfTI format. The first 10 volumes of each time series of echo-planar images were discarded to prevent the effect of the un-equilibrium of tissue magnetization that may introduce artifacts in the signal. The remaining 230 time points of each time course image series were used for analysis. The selected time course images were subsequently corrected for slice timing and realigned to the image of the first time point for the correction of the rigid-body head movement. At any time course, data exhibiting distortion from the movement larger than 1.5 mm and rotation larger than 1.5° were discarded. The time course images (3 mm × 3 mm × 3 mm) were then spatially normalized into the standard Montreal Neurological Institute space. Data were temporal band-pass filtered (0.01–0.08 Hz) to reduce the effects of low-frequency drift and physiological high-frequency noise. The linear trend was also removed.

The ReHo analysis was performed with the REST toolkit. The ReHo maps were generated for each subject by calculating the Kendall coefficient of concordance (KCC) of the time series of a given voxel. The KCC for each voxel in the brain was calculated voxel-wise by 27 voxels (one given voxel plus its adjacent 26 voxels). The standard ReHo value for each voxel is the KCC value of the voxel/the mean of the KCC values of the whole brain. Spatial smoothing was then performed on the standardized individual ReHo maps using an isotropic Gaussian filter with a Gaussian kernel of 6 mm and full-width at half maximum (FWHM).

### Statistical Analysis

For the demographic and clinical data, two-sample t-tests were conducted to identify the difference in age and education between the smokers and the controls. The threshold for all statistical significance was set at p<0.05.

For ReHo, two-sample t-test was performed to examine the voxel-wise difference between the smokers and the controls by the REST toolbox. Voxels with p<0.01 and cluster size >54 voxels were observed, which resulted in a corrected threshold of p<0.005 determined by AlphaSim (individual voxel P value = 0.01, FWHM = 6 mm, iterations = 1000, rmm = 5, with mask). These voxels were regarded as the regions of interest (ROI) showing significant difference between the two groups.

To further explore the brain region that may be related to the clinical variable of smokers, correlation analysis was performed between FTND and mean ReHo in a sphere with 5 mm radius centered on the location with the maximal t-value in the t-map in each ROI. In the analysis, p<0.05 was considered to be statistically significant.

## Results

### fMRI Results Based on ReHo

**Fig 1:**
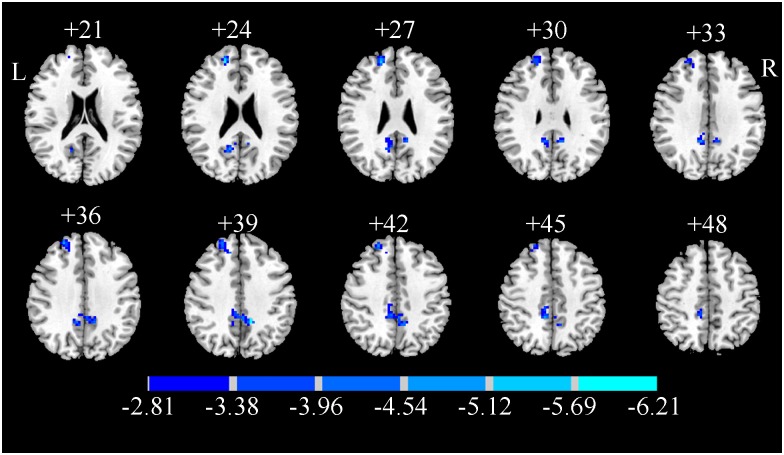
T-statistical difference maps between the smokers and the controls. Two-sample t-test, p<0.01, AlphaSim corrected.

The brain regions with significant difference in ReHo are shown in Fig 1 and Table 2. Two ROIs showed a significantly decreased ReHo values in the smokers compared with those in the controls. One ROI was located in the brain area of right superior frontal gyrus and right middle frontal gyrus in the Brodmann’s area (BA) 9. The other one was located in the left and right middle cingulum and the right precuneus in BA 23.

**Table 2:** ROIs of significantly different ReHo values between smokers and controls.

	Number of voxels	Brain area	BA	Peak t value	Peak coordinates (MNI)		
					x	y	z
1	78	superior frontal gyrus/R; middle frontal gyrus/R	9	−5.8927	18	48	24
2	143	precuneus/L, R;middle cingulum gyrus/L, R	23	−6.2106	−12	−45	39

R = right; L = left; BA = Brodmann’s area; MNI = Montreal Neurological Institute

### Correlation Result Between ReHo and FTND

In each ROI, the voxel with the maximum t-value was selected for further analysis. To examine the relationship between the clinical characteristics and the ReHo values in the ROI, correlation analysis was performed between FTND and mean ReHo in a sphere with 5 mm radius centered on the location with the peak voxel in each ROI.

**Fig 2:**
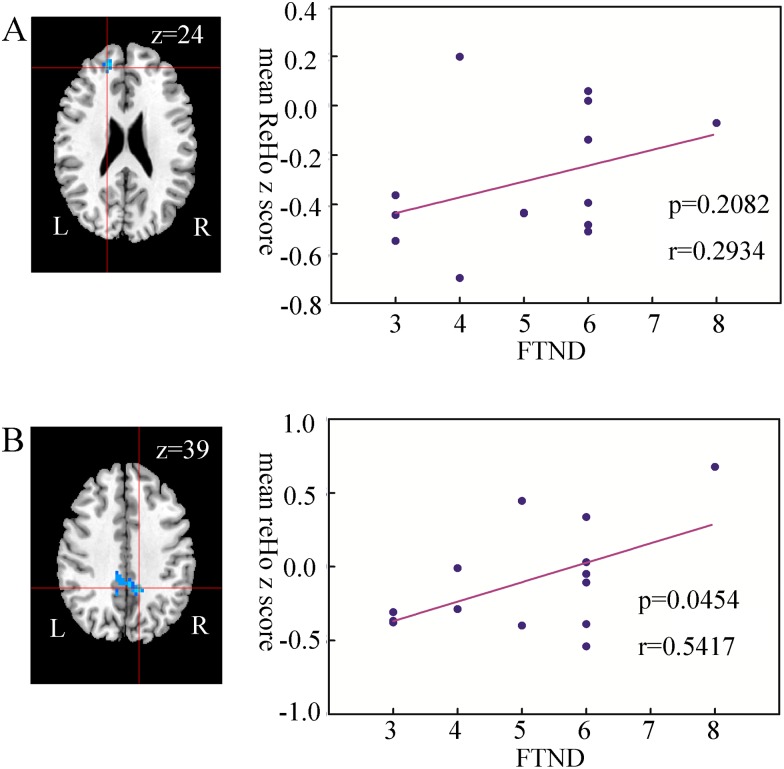
Results of correlation analysis between the FTND score and the ReHo value in smokers. A. The ReHo value in the superior frontal gyrus is insignificant and positively correlated with the FTND of the smokers (p>0.05, r = 0.2934); B. The ReHo value in the precuneus is significant and positively correlated with the FTND score of the smokers (p<0.05, r = 0.5417). FTND = Fagerstrom Test for Nicotine Dependence.

As shown in Fig 2, the ReHo values were positively related to the FTND scores in the brain regions. Moreover, the ReHo value in the precuneus was significantly and positively correlated with the FTND score ((p<0.05, r = 0.5417); however, no correlation was observed in the superior frontal gyrus (p>0.05, r = 0.2934).

## Discussion

In the present study, we conducted an rs-fMRI experiment to investigate the difference between smokers and non-smokers. In the experiment, 14 smokers and 11 non-smokers were scanned. The experimental results show that the ReHo value from the right superior frontal gyrus (BA 9) to the right middle frontal gyrus and that from the precuneus (BA 23) to the middle cingulum gyrus were lower in the smokers than in the non-smokers. The ReHo value in the precuneus (BA 23) was significant and positively correlated with the FTND score of smokers. These findings verify our hypothesis that the ReHo value of resting-state brain activity would be different between the smokers and the controls.

The rs-fMRI technology is concerned with the neuronal activity in the absence of a task and has been used to indirectly measure neuronal activity [25, 26]. As a principal method for rs-fMRI, ReHo is emerging as an interesting biomarker for measuring the means of diseases. The ReHo values in the right medial prefrontal-caudate circuit may represent an important biomarker of violence in juvenile males [27]. The ReHo abnormalities are a potential biomarker for the diagnosis of Parkinson’s disease, and they provide insights into the biological mechanism of the disease [28]. Zhan et al. experimentally demonstrated that ReHo may provide an objective biomarker for evaluating the functional abnormity of mild traumatic brain injury under acute settings [29]. Liu et al. proposed that ReHo is a potential neuroimaging biomarker for distinguishing bipolar disorder patients experiencing a depressive episode and unipolar depression patients [30]. Yuan et al. indicated that amnestic mild cognitive impairment patients show significant abnormal local coherence of biological activity in resting state, and ReHo can serve as a sensitive biomarker in functional imaging studies [31]. In addition, Chen et al. suggested that ReHo can be helpful in detecting minimal hepatic encephalopathy in cirrhotic patients [32]. The results of current study suggest that the ReHo values in the right superior frontal gyrus and left precuneus can be used to separate the smokers from the non-smokers. In particular, the left precuneus with the ReHo value that significantly correlates with the FTND score of smokers can be a potential neuroimaging biomarker for nicotine addicts.

The function for the brain areas of superior frontal gyrus and precuneus was related to the working memory in previous studies. A structural network research had shown that the precuneus, middle frontal gyrus, and superior frontal gyrus exhibit significantly high connectivity indices, which are remarkably related to variations in working memory capacity [33]. The repetitive transcranial magnetic stimulation has been navigated on the precuneus region, thereby resulting in a robust bilateral field advantage in visual short-term memory storage capacity [34, 35]. Systemic lupus erythematosus patients with memory impairment show significant hypoperfusion in the right precuneus compared with those with non-memory impairment; therefore, hypoperfusion of the precuneus may play a significant role in the memory function of systemic lupus erythematosus patients [36]. The results of the present study imply that the smokers are significantly different from the non-smokers in terms of working memory. Studying the particular cognitive function of nicotine addicts is thus important.

The current study has several limitations. First, our results are limited to a small sample size, which may affect the statistical analysis and comprehensive interpretation of the findings. Further studies with high number of patients of homogeneous etiology are needed, as well as high number of nicotine addicts. Second, the origin of ReHo and the neurophysiological basis remain unclear. These aspects must be further studied in the future.

In summary, we used ReHo based on rs-fMRI to explore the objective biomarker for nicotine dependence evaluation. The results show that the ReHo values in the right superior frontal gyrus and left precuneus can be used to separate the smokers from the non-smokers. Moreover, the ReHo value in the left precuneus is significantly correlated with the FTND score of smokers. These findings suggest that left precuneus is a potential neuroimaging biomarker for nicotine addicts.
